# Preoperative planning for implant placement with consideration of pelvic tilt in total hip arthroplasty: postoperative efficacy evaluation

**DOI:** 10.1186/s12891-016-1120-x

**Published:** 2016-07-13

**Authors:** Yutaka Inaba, Naomi Kobayashi, Haruka Suzuki, Hiroyuki Ike, So Kubota, Tomoyuki Saito

**Affiliations:** Department of Orthopaedic Surgery, Yokohama City University, 3-9 Fukuura, Kanazawa-ku, Yokohama, 236-0004 Japan

**Keywords:** Total hip arthroplasty, Preoperative planning, Pelvic tilt, Computer navigation, Implant position

## Abstract

**Background:**

In total hip arthroplasty (THA), tilting of the pelvis alters the cup placement angles. Thus, the cup angles need to be planned with consideration of the effects of pelvic tilt. In the present study, we evaluated the efficacy of preoperative planning for implant placement with consideration of pelvic tilt in THA, and the accuracy of a CT-based computer navigation for implant positioning.

**Methods:**

We examined 75 hips of 75 patients who underwent THA and were followed-up for one year postoperatively. The patients were divided into three groups (anterior, intermediate, posterior tilt) according to their preoperative pelvic tilt. Preoperative planning for implant placement was made with consideration of pelvic tilt and a CT-based navigation was used to execute the preoperative planning. Cup inclination, cup anteversion, and combined anteversion (CA) in supine and standing positions were examined 1 year after THA. The accuracy of the computer navigation was also examined.

**Results:**

Mean CA was 35.0 ± 5.8° in supine position and 39.3 ± 5.7° in standing position. CA did not differ among the three subgroups (anterior, intermediate, posterior tilt) in either supine or standing position, indicating implant placements to be equally effective. The desired CA (37.3°) was midway between those in supine and standing positions for each subgroup. Respective mean absolute errors between preoperative planning and postoperative CT measurement was 5.3 ± 5.2° for CA.

**Conclusion:**

We obtained favorable THA results with preoperative planning with consideration of pelvic tilt by demonstrating supine and standing CA to be unaffected by preoperative pelvic tilt one year postoperatively. Mean absolute error of CA between preoperative planning and postoperative measurement was 5° with use of the CT-based navigation.

## Background

In total hip arthroplasty (THA), the implant placement angle is associated with postoperative complications, such as wear of the articular surface and dislocation. It is also an important factor for achieving a favorable postoperative range of motion [1–3]. There are several reports on the optimum placement angle of the acetabular cup. Lewinnek et al. retrospectively studied cup placement angles in patients with a low rate of postoperative dislocation, reporting that the safe range consisted of cup inclination of between 30 and 50°, and cup anteversion of between 5 and 25° [4]. Kummer et al. reported that cup inclination of 35 to 45° and cup anteversion of 0 to 10° defined the optimum cup placement angle [5]. Moreover, Widmer et al. calculated an implant placement angle that would not cause implant impingement under strict conditions of the range of motion of the hip joint by using computer models, reporting that the conditions of ideal implant placement were cup inclination of 40 to 45°, and the sum of cup anteversion and stem antetorsion multiplied by 0.7 equaling 37.3° [6]. Yoshimine reported the results of a study based on the combined anteversion (CA) theory, in which cup anteversion and stem antetorsion are combined for consideration; this theory was adopted for this study [7].

Although these optimum cup placement angles are achieved in certain leg positions, tilting of the pelvis, to which the cup is fixed, alters the cup placement angles in tandem. Thus, the cup placement angles need to be planned with consideration of the effects of pelvic tilt. Lembeck et al. reported that cup anteversion changes by 0.7° for every 1° of pelvic tilt [8], while Babisch et al. also reported their study results showing that cup anteversion and cup inclination change by 0.8 and 0.3°, respectively, for every 1° of pelvic tilt [9]. According to these reports, if the pelvic tilt changes by 10°, cup anteversion will change by 7 to 8°, which cannot be ignored. Thus, several reports have indicated that it is important to plan cup placement with consideration of pelvic tilt before THA [10–15]. However, there are neither reports presenting specific preoperative planning methods, nor studies evaluating the usefulness of preoperative planning with consideration of pelvic tilt.

In the present study, we evaluated outcomes for postoperative implant placement angles in patients who underwent THA performed according to preoperative plans made with consideration of pelvic tilt.

## Methods

This prospective study was approved by our institutional review board (Yokohama City University, #B090507020) and all subjects provided informed consent. The subjects were 75 patients (75 hips) who underwent THA performed with computer navigation according to preoperative plans made with consideration of pelvic tilt, and who could be followed up for at least 1 year after surgery. There were 57 women and 18 men, with a mean age of 64.3 years (38–89 years) at the time of surgery. The primary diseases were secondary hip osteoarthritis due to developmental dysplasia of the hip in 62 hips, osteonecrosis of the femoral head in 11 hips, and rheumatoid arthritis in two hips. The present study excluded patients with bilateral lesions and included only those with a unilateral lesion. In all patients, the procedure was performed in the lateral decubitus position through the anterolateral approach with a computed tomography (CT)-based navigation system (Stryker Inc., Kalamazoo, MI, USA).

In all patients, anteroposterior (AP) pelvic radiographs (in the supine and standing positions) were performed before and 12 months after surgery, and plain pelvic CT was performed before and 1 week after surgery. In AP pelvic radiographs, the center of the X-ray image was aligned with the upper border of the pubic symphysis. According to a report by Nishihara et al. [16], we reproduced conditions in the supine and standing positions with 3-dimensional models reconstructed from pelvic CT images. To make the aspect ratio of the pelvic cavity on an AP pelvic radiograph equal to the ratio in a 3-dimensional reconstructed CT model, the model was rotated along the horizontal axis. The inclination of the anterior pelvic plane (APP), as defined by both anterior superior iliac spines and pubic tubercles [10] relative to the vertical plane, was recorded as a positive value. Lateral radiograph of the lumbar spine was performed in the standing position before surgery, and the lumbolordotic angle was measured. The lumbolordotic angle was defined as an angle formed between the lower margin of the 12th thoracic vertebral body and the upper margin of the first sacral vertebral body, and measured as a positive value.

For preoperative planning, navigation software was used to first plan stem antetorsion, and then to plan cup placement angles according to stem antetorsion. On the basis of the report by Widmer et al. [6], cup anteversion was planned to obtain an angle of 37.3°, as derived from the equation, cup anteversion + stem antetorsion × 0.7, and the target cup inclination was set at 40°. A problem in this process is which plane to use as a reference for cup placement. In many patients, we used the preoperative supine APP as a functional pelvic plane (FPP) for preoperative planning. However, in patients with preoperative anterior pelvic tilt exceeding 10° in the standing position (anterior tilt group), the target reference plane was set at −10° from the standing APP on the basis of a result showing that the pelvis tilts backward by 10° on average after THA [17]. Meanwhile, in patients with the lumbolordotic angle reduced to 30° or less by the effects of lumbar compression fractures and the like among those with a posterior preoperative pelvic tilt of −10° or less in the standing position (posterior tilt group), the midpoint between the supine and standing APPs was used as the FPP for preoperative planning, on the basis of a result showing that the pelvis tilts further backward after THA [18]. In patients with preoperative pelvic tilt between −10 and 10° in the standing position (intermediate group) and those in the posterior tilt group with the lumbolordotic angle exceeding 30°, the preoperative supine APP was used as the FPP for preoperative planning (Table 1).

**Table 1:** The reference pelvic planes used for preoperative planning

1. Anterior tilt group (preoperative standing APP > 10°): Reference, standing APP −10°	9 hips
2. Intermediate group (−10° < preoperative standing APP ≤ 10°): Reference, supine APP	45 hips
3. Posterior tilt group (preoperative standing APP ≤ −10°)	21 hips
LLA > 30°: 11 hips; reference, supine APP	
LLA ≤ 30°: 10 hips; reference, the midpoint between the supine and standing APPs	

*APP* anterior pelvic plane, *LLA* lumbolordotic angle

In the present study, THA was performed with intraoperative assistance using CT-based navigation according to these preoperative plans. Cup and stem placement angles and CA values in the supine and standing positions were examined 1 year later. Cup inclination and cup anteversion were expressed as radiographic angles, while CT measurements were used for stem antetorsion. The CA value was calculated from the following equation according to the report by Widmer et al.: cup anteversion + stem antetorsion × 0.7 [6]. To determine to what extent the preoperative plans were actually executed during surgery, the accuracy of computer navigation and the accuracy of execution of preoperative plans were evaluated. To determine the accuracy of computer navigation, absolute values of differences between intraoperative navigation measurements and postoperative CT measurements were calculated as absolute errors. For the accuracy of execution of preoperative plans, the absolute values of differences between preoperatively planned values and postoperative CT measurements were calculated. In addition, regarding postoperative complications, the incidence of dislocation was assessed.

Statistical analysis was performed by the Mann-Whitney *U* test for comparison between the groups. A *P* value of less than 0.05 was considered to indicate a significant difference.

## Results

In the present study including 75 hips, the anterior tilt group, with preoperative pelvic tilt exceeding 10° in the standing position, included 9 hips (12 %), and the intermediate group, with preoperative standing APP values between −10 and 10° included 45 hips (60 %). The posterior tilt group, with preoperative pelvic tilt of −10° or less in the standing position, included 21 hips (28 %). The lumbolordotic angle was 30° or less in 10 hips in the posterior tilt group (13 %) and exceeded 30° in 11 hips (15 %) (Table 1).

At 1 year after surgery, implant placement angles in the supine and standing positions were measured. Overall, in the supine position the mean cup inclination was 39.3 ± 4.1°; the mean cup anteversion was 16.9 ± 6.0°, and the mean CA was 35.0 ± 5.8°. In the standing position, the mean cup inclination was 40.5 ± 4.2°; the mean cup anteversion was 20.8 ± 6.3°, and the mean CA was 38.9 ± 5.7°. The mean CA in the supine position was 36.0 ± 4.5° in the anterior tilt group, 35.6 ± 5.9° in the intermediate group, and 34.1 ± 6.0° in the posterior tilt group, showing no significant difference between the three groups. The outcomes of implant placement were comparable in all groups. Moreover, the CA values in the standing position showed no difference between the three groups. In any group, the ideal CA of 37.3° was situated at the midpoint between CA in the supine and standing positions (Table 2).

**Table 2:** Implant placement angles at 1 year after surgery

Supine	Number	Cup inclination	Cup anteversion	Stem antetorsion	CA
Overall	75	39.3 (4.1)	16.9 (6.0)	25.8 (8.7)	35.0 (5.8)
Anterior tilt	9	38.1 (3.3)	16.3 (9.3)	28.1 (10.1)	36.0 (4.5)
Intermediate	45	39.1 (4.1)	17.4 (5.6)	26.1 (8.8)	35.6 (5.9)
Posterior tilt	21	39.9 (4.4)	16.2 (5.5)	24.1 (8.1)	34.1 (6.0)
Standing	Number	Cup inclination	Cup anteversion	Stem antetorsion	CA
Overall	75	40.5 (4.2)	20.8 (6.3)	25.8 (8.7)	38.9 (5.7)
Anterior tilt	9	39.5 (3.6)	20.8 (8.2)	28.1 (10.1)	40.5 (5.0)
Intermediate	45	40.1 (4.3)	20.0 (6.0)	26.1 (8.8)	38.2 (5.9)
Posterior tilt	21	42.1 (4.1)	22.6 (5.4)	24.1 (8.1)	39.5 (5.6)

*CA* combined anteversion. The figures in parentheses are standard deviations. The measurements are expressed as radiographic angles

Table 3 shows intraoperative navigation measurements and postoperative CT measurements determined with an APP value of 0° for cup inclination, cup anteversion, stem antetorsion, and CA. Although no significant difference was observed between the intraoperative navigation measurements and the postoperative CT measurements, the absolute differences between them were 2.3 ± 2.1° for cup inclination, 2.1 ± 2.0° for cup anteversion, 3.0 ± 2.4° for stem antetorsion, and 3.5 ± 3.2° for CA. Meanwhile, although no significant difference was observed between the preoperatively planned values and postoperative CT measurements, the absolute differences between them were 3.5 ± 2.6° for cup inclination, 4.0 ± 3.5° for cup anteversion, 3.9 ± 5.0° for stem antetorsion, and 5.3 ± 5.2° for CA (Table 4). Postoperative dislocation was not observed in any patients during follow-up period.

**Table 3:** Accuracy of implant placement

	Cup inclination	Cup anteversion	Stem antetorsion	CA
Intraoperative navigation measurement (°)	39.7 (3.7)	17.3 (6.7)	25.2 (8.5)	34.9 (6.5)
Postoperative CT measurement (°)	39.2 (4.2)	16.9 (6.3)	25.8 (8.7)	34.6 (7.7)
*P* value	0.36	0.58	0.67	0.80
Absolute error (°)	2.3 (2.1)	2.1 (2.0)	3.0 (2.4)	3.5 (3.2)

*CA* combined anteversion. The figures in parentheses are standard deviations. The intraoperative navigation measurements and postoperative CT measurements are expressed as radiographic angles relative to an anterior pelvic plane value of 0°

**Table 4:** The accuracy of executing preoperative plans

	Cup inclination	Cup anteversion	Stem antetorsion	CA
Preoperatively planned value (°)	39.4 (1.4)	16.3 (6.3)	26.2 (8.1)	34.4 (7.2)
Postoperative CT measurement (°)	39.2 (4.2)	16.9 (6.3)	25.8 (8.7)	34.6 (7.7)
*P* value	0.57	0.40	0.82	0.62
Absolute error (°)	3.2 (2.3)	4.0 (3.5)	3.9 (5.0)	5.3 (5.2)

*CA* combined anteversion. The figures in parentheses are standard deviations. The preoperatively planned values and postoperative CT measurements are expressed as radiographic angles relative to an anterior pelvic plane value of 0°

## Discussion

**Fig. 1:**
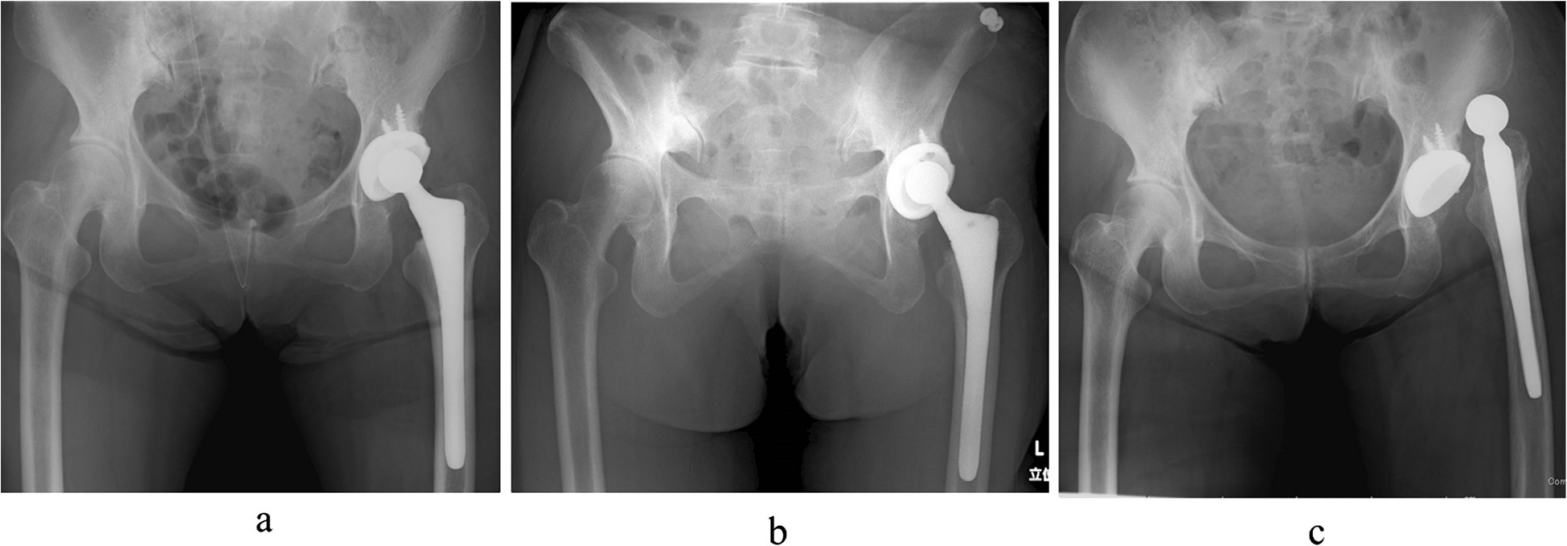
A 64-year-old woman with left hip osteoarthritis (after total hip arthroplasty). Anteroposterior pelvic radiographs taken after left total hip arthroplasty (THA) are shown. In the supine position (**a**), cup inclination is 45°, and cup anteversion is 25°. However, in the standing position, the pelvis markedly tilts backward; cup inclination is 59°, and cup anteversion is 42° (**b**). Although this case is not included in the present study, the case, in which the hip joint was anteriorly dislocated 2 weeks after THA (**c**), triggered the initiation of the study

In clinical practice, there are cases in which, although favorable cup placement angles are seen on plain radiographs taken in the supine position after THA, the pelvis substantially tilts backward in the standing position so that cup inclination and cup anteversion markedly deviate from the safe range (Fig. 1). In recent years, the effects of pelvic tilt on cup placement angles have attracted attention, and there are sporadic reports on fluctuations in pelvic tilt along with posture changes, such as between the supine and standing positions, and on changes in pelvic tilt after THA [19–21]. These reports emphasize that meticulous attention needs to be paid to implant placement during THA in patients with pelvic tilt substantially changing with postures or after surgery. However, there has been no report specifically indicating which degree of cup placement angle should be aimed for in different types of patients, while no study on the usefulness of planning of implant placement with consideration of pelvic tilt has been reported.

Thus, we aimed, in the present study, to assess the usefulness of our method of preoperative planning with consideration of pelvic tilt. According to the results of our previous studies [17, 18], in all patients with marked preoperative anterior pelvic tilt in the standing position (anterior tilt group), the pelvis tilted backwards after THA, and the mean retroversion was 10° at 1 year after surgery. These patients were relatively young with developmental dysplasia of the hip. Fujii et al. [22] reported that the pelvis tilted more anteriorly in dysplastic hips than in controls. We believe that improvements in anterior pelvic tilt might occur after THA in patients with dysplasia of the hip. The elimination of preexisting flexion contractures of the hip joint might also cause postoperative retroversion after THA. Thus, in patients with marked anterior pelvic tilt, the target angles were planned by subtracting 10° from the preoperative standing APP value. Moreover, in the intermediate group with preoperative standing APP values between −10 and 10°, the target was set at the preoperative supine APP because changes in pelvic tilt after THA were small. The preoperative supine APP was used as a reference because the supine position is the most stable leg position in which CT is performed [16]. In patients with marked posterior pelvic tilt, defined as a preoperative standing APP tilting by −10° or less (posterior tilt group), changes in pelvic tilt after THA are largely divided into two patterns: patients with little change, and patients with progression of posterior pelvic tilt after THA. The latter group of patients requires a high degree of caution in preoperative planning. They were characterized by a decrease in lumbar lordosis. In patients with lumbolordotic angles decreasing to 30° or less in the posterior tilt group, the targeted reference plane was shifted toward posterior tilt during preoperative planning, and the midpoint between the preoperative supine and standing APPs was targeted. The midpoint was targeted based on the results of our previous studies showing that the pelvis tilted more backward in the standing position than in the supine position in all patients with posterior pelvic tilt, and that the mean supine APP value at 1 year after THA was the midpoint between the preoperative supine and standing APP values [17, 18]. In addition, the cutoff value for decreased lumbolordotic angle was set at 30° in the present study. This value was decided upon based on the results of our previous study showing that the mean lumbolordotic angle was 23.3 ± 17.1° in patients with further progression of posterior pelvic tilt after THA in the posterior tilt group [18], on the fact that the mean lumbolordotic angle is 53.6 ± 9.9° in the Japanese population [23], and on the value derived from the subtraction of two standard deviations (SDs) from this mean which is approximately 30°. Legaye et al. [24] examined a normal population of 49 adults without vertebral disease, and reported that the lumbar lordosis angle was 61.4 ± 10.2° for men and 58.1 ± 10.8° for women. Roussouly et al. [25] reported that the lumbar lordosis angle was 61 ± 9.7° in 154 asymptomatic adults. Considering these values in normal European populations, the cutoff value, 30° of lumbolordotic angle, might be considered appropriate.

**Fig. 2:**
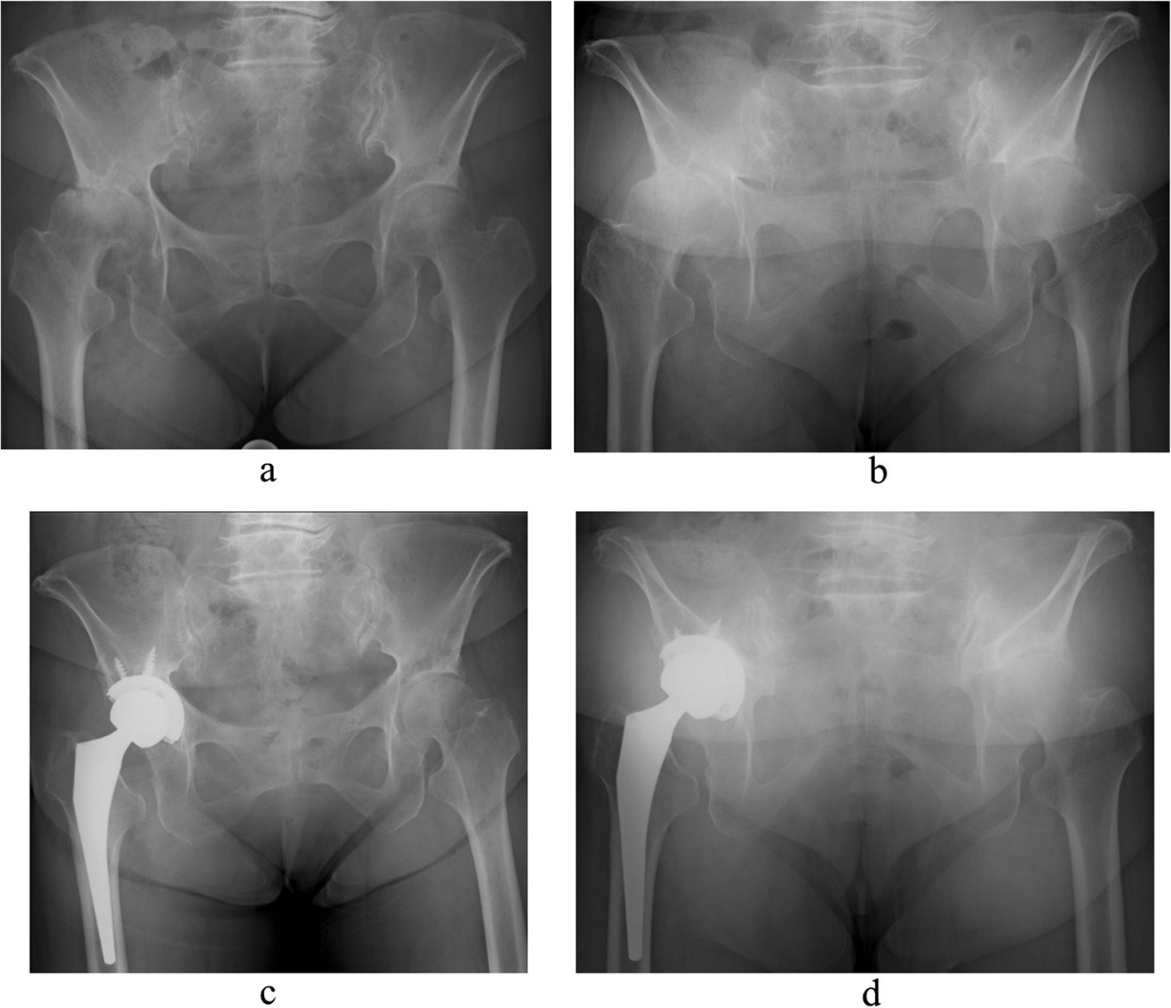
A 70-year-old woman with right hip osteoarthritis. On plain pelvic radiographs taken before right total hip arthroplasty (THA), the pelvis tilts backward by 18° in the supine position (**a**) and 34° in the standing position (**b**). Because the lumbolordotic angle had decreased to 5° in this case, the target reference plane for preoperative planning was set at retroversion of 30°. On radiographs taken at 1 year after THA, the pelvis tilts backward by 24° in the supine position (**c**) and 38° in the standing position (**d**), and combined anteversion (CA) is 31.3° in the supine position and 39.2° in the standing position. Because the ideal CA value of 37.3° is situated between the CA values in the supine and standing positions, the pelvic tilt is less likely to deviate from 37.3° even with further progression of posterior pelvic tilt in the future

When THA was performed according to the plans described above, the implant placement angles in the supine and standing position at 1 year after surgery did not differ between the anterior, intermediate, and posterior tilt groups, and comparable implant placement was achieved regardless of preoperative pelvic tilt. Moreover, the 37.3° of CA that we aimed for was situated almost at the midpoint between CA in the supine and standing positions at 1 year after surgery. When the posture was changed from the supine to standing position or vice versa, pelvic tilt fluctuated from the ideal value of 37.3° at the center. Thus, it is assumed that the tilt was less likely to greatly deviate from 37.3° even with these changes in the posture, and that safe and favorable implant placement had been performed (Fig. 2).

However, even when an ideal preoperative plan is made, it is worthless without execution of the plan in surgery. Thus, we used computer navigation. However, the error between the preoperatively planned values and postoperative placement angles was approximately 5° in terms of CA. One of the reasons for this error in the present study is that even a difference of 2 to 3° between intraoperative implant placement angles and preoperatively planned values was tolerated. For example, when the cup was intraoperatively placed by press-fit at 17° instead of the preoperatively planned cup anteversion of 20°, the error was tolerated. In such cases, the preoperative plan was often revised intraoperatively by inserting the stem with the antetorsion angle increased by a few degrees. Given previous reports indicating that the errors in implant placement with CT-based navigation are 2 to 3° for the cup and 3 to 4° for the stem [26, 27], this error of 5° appears to be valid. However, because the SD is large at approximately 5°, a future issue is how much this error can be reduced. In addition, because even the use of computer navigation causes an error of approximately 5°, it should be realized that manual operation without computer navigation is highly likely to cause even larger errors.

The limitations of the present study include the lack of assessment of values in the sitting position. Although the pelvis tilts backward in the sitting position, flexion of the hip joints and changes in the flexion angle of the hip joints seen in the sitting position make assessment difficult. As only values obtained in the supine and standing positions were assessed, the present study ideally needs detailed assessment based on motion analysis and the like. For evaluation of the effects of pelvic tilt on THA, another problem is imposed by the presence of patients with a great difference between pelvic tilt in the supine and standing positions. Nishihara et al. reported that patients with a difference between supine and standing APP values of more than 10° accounted for approximately 10 % of patients [16]. In the present study, a difference between supine and standing APP values by more than 10° was observed in ten patients (13.0 %) before surgery but in three patients (3.7 %) at 1 year after surgery. A large-diameter head was used to treat hips with a large difference between pelvic tilt in the supine and standing positions, and dislocation was not observed after surgery. Moreover, the results of the present study suggest that problematic cases are limited because the difference between supine and standing APP becomes smaller at 1 year after THA. However, further studies on preoperative planning in such cases are needed in the future.

The present study was conducted on only patients with a unilateral lesion. The reason for this is that those with bilateral lesions cannot accurately be assessed because the change in pelvic tilt after THA for the hip joint on one side will be affected by pain, flexion contracture, and other symptoms of the hip joint at the opposite side. Patients with different pathologies, including osteoarthritis of the hip and osteonecrosis of the femoral head, were included in the present study. We believe that the influence of differing pathologies on postoperative pelvic movement would be reduced by exclusion of bilateral cases, because all patients would have a healthy hip on the contralateral side after THA. Moreover, the reference used for preoperative planning in the present study is still provisional. Detailed analyses and studies need to be conducted with a larger sample size in the future.

## Conclusions

When THA was performed according to preoperative plans made with consideration of preoperative pelvic tilt, CA in the supine and standing positions at 1 year after surgery was not affected by preoperative pelvic tilt, showing favorable values. Even with the use of computer navigation, a tool for the accurate execution of preoperative plans, the error in CA from the preoperatively planned values was approximately 5°.

## Abbreviations

APP, anterior pelvic plane; CA, combined anteversion; CT, computed tomography; FPP, functional pelvic plane; THA, total hip arthroplasty
